# Longitudinal changes in telomere length and associated genetic parameters in dairy cattle analysed using random regression models

**DOI:** 10.1371/journal.pone.0192864

**Published:** 2018-02-13

**Authors:** Luise A. Seeker, Joanna J. Ilska, Androniki Psifidi, Rachael V. Wilbourn, Sarah L. Underwood, Jennifer Fairlie, Rebecca Holland, Hannah Froy, Ainsley Bagnall, Bruce Whitelaw, Mike Coffey, Daniel H. Nussey, Georgios Banos

**Affiliations:** 1 Animal & Veterinary Sciences, SRUC, Easter Bush, Midlothian, United Kingdom; 2 The Roslin Institute and Royal (Dick) School of Veterinary Studies, University of Edinburgh, Easter Bush, Midlothian, United Kingdom; 3 Royal Veterinary College, University of London, Hatfield, United Kingdom; 4 Institute of Evolutionary Biology, School of Biological Sciences, University of Edinburgh, Edinburgh, Midlothian, United Kingdom; 5 SRUC Crichton Royal Farm, Dumfries, United Kingdom; Centre National de la Recherche Scientifique, FRANCE

## Abstract

Telomeres cap the ends of linear chromosomes and shorten with age in many organisms. In humans short telomeres have been linked to morbidity and mortality. With the accumulation of longitudinal datasets the focus shifts from investigating telomere length (TL) to exploring TL change within individuals over time. Some studies indicate that the speed of telomere attrition is predictive of future disease. The objectives of the present study were to 1) characterize the change in bovine relative leukocyte TL (RLTL) across the lifetime in Holstein Friesian dairy cattle, 2) estimate genetic parameters of RLTL over time and 3) investigate the association of differences in individual RLTL profiles with productive lifespan. RLTL measurements were analysed using Legendre polynomials in a random regression model to describe TL profiles and genetic variance over age. The analyses were based on 1,328 repeated RLTL measurements of 308 female Holstein Friesian dairy cattle. A quadratic Legendre polynomial was fitted to the fixed effect of age in months and to the random effect of the animal identity. Changes in RLTL, heritability and within-trait genetic correlation along the age trajectory were calculated and illustrated. At a population level, the relationship between RLTL and age was described by a positive quadratic function. Individuals varied significantly regarding the direction and amount of RLTL change over life. The heritability of RLTL ranged from 0.36 to 0.47 (SE = 0.05–0.08) and remained statistically unchanged over time. The genetic correlation of RLTL at birth with measurements later in life decreased with the time interval between samplings from near unity to 0.69, indicating that TL later in life might be regulated by different genes than TL early in life. Even though animals differed in their RLTL profiles significantly, those differences were not correlated with productive lifespan (p = 0.954).

## Introduction

Telomeres are located at the ends of linear chromosomes. They consist of non-coding nucleotide tandem repeats (TTAGGG in vertebrates) and attached proteins of the shelterin complex [1–3]. Since telomeres were first shown to shorten with the number of cell divisions in vitro [4], they have been intensely studied in relation to ageing and lifespan in various species in vivo [5–9]. Such studies have reported mixed results. While some observed a positive correlation between telomere length and longevity [5,10–12], others found no relationship [13,14]. Many authors claimed that longitudinal studies were necessary to better understand telomere dynamics within the individual, and to investigate the association of not only telomere length but also change in telomere length with lifespan [10,15–17]. In longitudinal studies of Alpine swifts and Seychelles warblers, faster telomere attrition, but not telomere length per se, was associated with poorer survival [18,19]. In humans telomere length maintenance was associated with better survival than telomere length attrition in patients with cardiovascular disease [20,21]. However, the relationship between telomere length attrition and survival has not been investigated in a livestock species to date.

Genetic studies on telomere length are rare outside the human literature. In humans it has been shown that telomere length is a quantitative trait that is controlled by many different loci [22–26]. Heritability estimates are available for humans, sand lizards and kakapos and range from 0.39 to 0.82 in those species [27–33]. Outside those studies heritability estimates are missing from the literature. It has been shown in the above mentioned species that telomere length is a heritable trait, but it is unclear if heritability estimates change over life or are relatively constant. A changing impact of environmental effects on telomere length might change heritability estimates over time. For animal breeders it is interesting to know which proportion of a trait at any time is caused by genetic effects and therefore possible to influence with breeding.

In the livestock sector there is a growing interest in using telomere length as a biomarker for health, productive lifespan and animal welfare [34,35]. However, longitudinal studies that investigate change in telomere length within individuals are largely missing from the livestock literature. In the present study we are interested in the rate and direction of telomere length change and the relationship of different telomere length change profiles with productive lifespan. We use random regression models which were initially developed to describe lactation curves in dairy cattle [36,37] for the analysis of telomere length profiles. They allow the fitting of an overall fixed curve across time which describes the population trend, and individual random animal curves (profiles) as deviations from the former. Random regression models take into account the correlation among repeated measurements within an individual, which is usually greater than the correlation of measurements between animals [38]. Over the last two decades random regression models have been applied to many studies in genetics and evolutionary ecology addressing the change of a broad range of traits over time. Examples of studied traits in genetics include milk yield [39], milk fat and protein content [40], somatic cell count [41], body condition score [42,43], body energy [44] and carcass traits [45]. In evolutionary ecology studied traits included fitness [46], body size [47], body weight in relation to faecal egg counts [48] and antler size [49]. To our knowledge, only a single study has used random regression models for the analysis of longitudinal telomere data so far [18]. However, the study was based on a rather small dataset (373 samples of 204 individuals; more than half of the individuals were sampled once only) and could not find a statistically significant difference in telomere length profiles.

The objectives of the present study were to 1) characterize the change in bovine relative leukocyte telomere length (RLTL) across the lifetime in Holstein Friesian dairy cattle, 2) estimate genetic parameters of RLTL over time and 3) investigate the association of differences in individual RLTL profiles with productive lifespan.

## Materials and methods

### Ethics statement

Blood sampling of Holstein Friesian cattle was approved by the Animal Experiments Committee (UK Home Office Project License Number: PPL 60/4278).

### Data

Animals used in this study were Holstein Friesian dairy cattle of the Langhill herd that were kept at the Crichton Royal Research Farm in Dumfries (Scotland, UK). All animals in this herd belong to one of two distinct genetic lines (selected for high milk fat and protein yield vs. control). Furthermore, cows are randomly allocated to two different diets that contain either a high or low proportion of forage. These genetic lines and diets were set up over 30 years ago to accommodate genetic and nutritional scientific studies [50].

We measured RLTL in 1,328 longitudinal samples of 308 female animals born between 2008 and 2014. Animals were approximately equally split between genetic lines and diets. All animals were blood sampled once at birth and then at least once more during their lifetime. On average, 4.3 samples were taken per animal. At the end of the study 244 out of 308 animals were dead and had recorded productive lifespan measurements. Productive lifespan was defined as the time between the animal’s birth and culling in days. Productive lifespan differs from longevity measurements in humans and natural populations, because dairy cattle rarely die of natural causes. However, we argue that productive lifespan is still biologically meaningful, because animals are not culled randomly but usually for fertility or health reasons.

DNA was extracted from whole blood samples using DNeasy spin columns (QIAGEN) and each sample had to pass internal quality control steps which were 1) yield and purity measured on a NanoDrop ND-1000 spectrophotometer (Thermo Scientific) had to fulfil the minimum requirements of: yield > 20 ng/μl, 260/280 ratio > 1.7 and 260/230 ratio >1.8 and 2) integrity gel scores had to be between 1–2 [51]. RLTL was measured by qPCR using tel 1b (5’-CGG TTT GTT TGG GTT TGG GTT TGG GTT TGG GTT TGG GTT-3’) and tel 2b (5’-GGC TTG CCT TAC CCT TAC CCT TAC CCT TAC CCT TAC CCT-3’) primers [52] for the telomere amplification and beta-2-microglobulin (B2M) primers (Primerdesign, accession code NM_001009284) for the reference gene amplification [51]. An identical sample–the so-called calibrator or golden sample–was repeated on every plate to correct for measurement error that is associated with the qPCR plate. The number of cycles at which the qPCR amplification curve crosses a set fluorescence threshold (the Cq value) was determined for each sample for telomere and B2M reactions. Raw Cq measurements were baseline corrected using the software LinReg PCR [53]. The same software was used to calculate the reaction specific qPCR efficiencies E_TEL_ and E_B2M_ that were in turn used in following formula [54] to calculate RLTL:

$$RLTL=\frac{E_{TEL}^{{Cq}_{TEL(Calibrator)}-{Cq}_{TEL(Sample)}}}{E_{B2M}^{{Cq}_{B2M(Calibrator)}-{Cq}_{B2M(Sample)}}}$$ (1)

The Cq values corresponding with the calibrator sample were Cq_TEL(Calibrator)_ and Cq_B2M(Calibrator)_ for the telomere and the B2M reaction respectively. Cq values of the individual samples were Cq_TEL(Sample)_ and Cq_B2M(Sample)_.

Individual samples were measured on 25 qPCR plates in total which had 8 rows for each reaction. RLTL data were logarithmically transformed to achieve normal distribution (Shapiro-Wilk normality test: W = 0.9985, p = 0.299). Because of the increasing scarcity of data points after the age of 60 months, this age was used as the cut-off for data visualisation. The pedigree included 11,003 animals spread over 27 generations. The animals with RLTL measurements were descendants of 40 sires and 241 dams.

### Data analysis

The following random regression model was used for the analysis of longitudinal RLTL data:

$$Y_{tijk}={BirthYear}_{j}+{GeneticGroup}_{j}+{qPCRplate}_{ij}+{qPCRrow}_{ij}+\sum_{k=0}^{n}{P_{jkt}b}_{k}+\sum_{k=0}^{n}P_{jkt}u_{jk}+e_{tijk}$$ (2)

where Y_tijk_ = the i^th^ RLTL measurement for animal j using a Legendre polynomial of the order k. BirthYear_j_ represents the fixed effect of the year in which animal j was born; GeneticGroup_j_ stands for the fixed effect of the genetic group of animal j; qPCR plate and qPCR row of a particular sample i of animal j was included as fixed effects (qPCRplate_ij_ and qPCRrow_ij_); fixed effects regression coefficients are represented byb_k_, while u_jk_ stands for the k^th^ order random regression coefficients for the additive genetic effects of animal j; P_jkt_ represents the kth order of Legendre polynomial fitted to the measurement i of animal j at the age t in months; the random residual variance is e_tijk_. Sampling intervals and age at sampling (after the initial record) differed among individuals.

Model (2) included fixed effects that remained statistically significant (p<0.05) after backwards eliminating all tested non-significant effects (such as birth season, birth weight, weight at sampling, body condition score, and feed group) and the genetic group of the animal. The fixed and random regressions, both modelled with polynomial functions, described the average RTL change across age, and individual animal deviations from the average, respectively. The latter pertained to the animal’s additive genetic effect. The animal’s permanent environment was also examined as a random factor but had a negligible effect (see S1 File).

We tested if the residual variance of different age groups differed significantly implying a heterogeneous variance structure. We first considered four different age groups (0–12 months, 13–24 months, 25–40 months and older than 40 months) and then two different age groups (younger and older than 2 months) but did not find a significant difference in residual variance between any age groups (see S1 File). Therefore, a homogeneous residual variance structure was assumed for the subsequent analysis.

The Akaike information criterion (AIC) was used to assess 1) if the introduction of the random animal genetic effect improved the model fit compared to a model that only included fixed effects; this would suggest that animals differ in their intercept (average RLTL across all measurements); 2) if Legendre polynomials fitted to the random animal genetic effect improved the model fit further, thereby suggesting that animals also differ in their slope (RLTL dynamics). A difference of two units in AIC corresponds to an approximate significance of p<0.05. Within the range of two units the simpler model was preferred over the more complicated [55–57]. In the end, quadratic polynomials were fitted to both the overall fixed curve and the individual random animal deviation.

All statistical analyses were conducted with the ASReml software version 4.1 [58].

#### Calculation of the fixed and random curves

The fixed curve that illustrates RLTL dynamics at a population level was calculated as the sum of the products of the Legendre polynomial order residuals for a given age and the corresponding fixed regression coefficients. This was repeated across all ages in the trajectory. Random regression models allow the calculation of an individual profile of RLTL change over age for each animal as a deviation from the population mean (fixed curve). The model output provides estimates (solutions) for each animal and each order of polynomial fitted in the model. The random curves were calculated simply by summing solutions for each animal and test month across all products of the n^th^ order polynomial with the n^th^ order polynomial residual. The standard error was calculated in parallel by using the standard errors associated with the solutions for the same calculation. Eigenvalues were calculated to estimate the amount of variance between animals that is due to 1) the intercept and 2) the shape of individual curves. Eigenfunctions were calculated to analyse the direction of each effect.

#### Variance components and genetic parameters

The additive genetic variance (V_A_) for each month was calculated using following formula [38]:

$$V_{A}=pKp\prime$$ (3)

Where p is a 1 x k vector (k is the order of the fitted Legendre polynomial) containing the residuals for each polynomial order for the given month, K is a matrix containing the REML estimates of (co)variance components and p’ is the transposed p vector. The heritability of RLTL and its standard error were calculated at birth and for each consecutive month. Also, the genetic correlations of RLTL at birth with each following month were calculated. Detailed information about those calculations can be found in S1 File.

#### Analysis of the association of RLTL dynamics with productive lifespan

Out of 308 animals 244 were dead by the end of the study and produced exact productive lifespan measurements. To investigate if different RLTL profiles were associated with a difference in productive lifespan, individual RLTL random curves (profiles) were clustered using the R library kmlShape [59] in five groups. We decided for five clusters to explore a difference in animals that maintain their RLTL in contrast to those who early in life either mildly or moderately shorten or elongate their RLTL, respectively. The association between productive lifespan and RLTL cluster was investigated with a Cox proportional hazard analysis. This analysis allows fitting maximal known survival times as right-censored data to account for animals that are still alive. For living animals age in days at the first day of the present year was used for the calculation of the maximal known survival time. A Wald test was used to determine the significance of the relationship between RLTL profiles and productive lifespan.

## Results

Raw RLTL measures ranged from 0.693 to 1.727 with a mean of 1.082. The coefficient of variation was 0.162. The model that included the animal identity as a random effect fitted the data significantly better than a model including only the fixed effects (delta AIC = 204.97) suggesting that animals differed significantly in their average RLTL across time. Fitting animal identity with pedigree information further improved the model fit (delta AIC = 55.46). Fitting an individual curve for each animal (using a quadratic Legendre polynomial) additionally increased the model fit (delta AIC = 3.24), meaning that monthly RLTL dynamics also differ among individual animals. A quadratic Legendre polynomial fitted marginally better than a linear function (delta AIC = 2.07) and had the advantage that the same order of Legendre polynomial was fitted to the fixed and the random effect which facilitates interpretation of the results.

**Fig 1:**
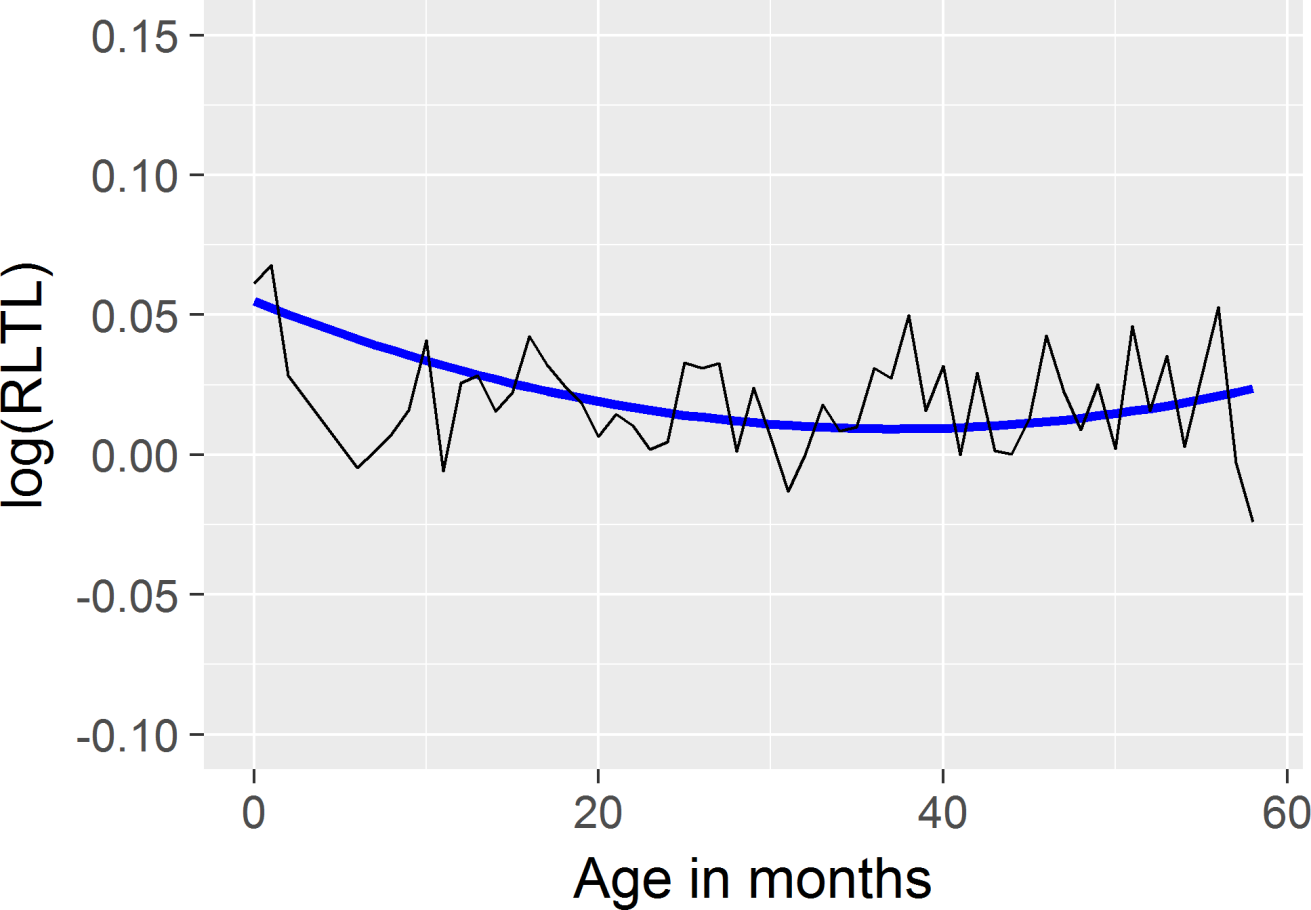
Fixed curve of logarithmically transformed relative leukocyte telomere (RLTL) data. Blue line: quadratic Legendre polynomial function of age; black solid line: phenotypic RLTL measurements for each month.

The fixed curve as described by the Legendre polynomial captured the expected initial decline of RLTL in early life and a relative stability of RLTL later in life (Fig 1). The curve also illustrates a slight increase of RLTL in later life.

**Fig 2:**
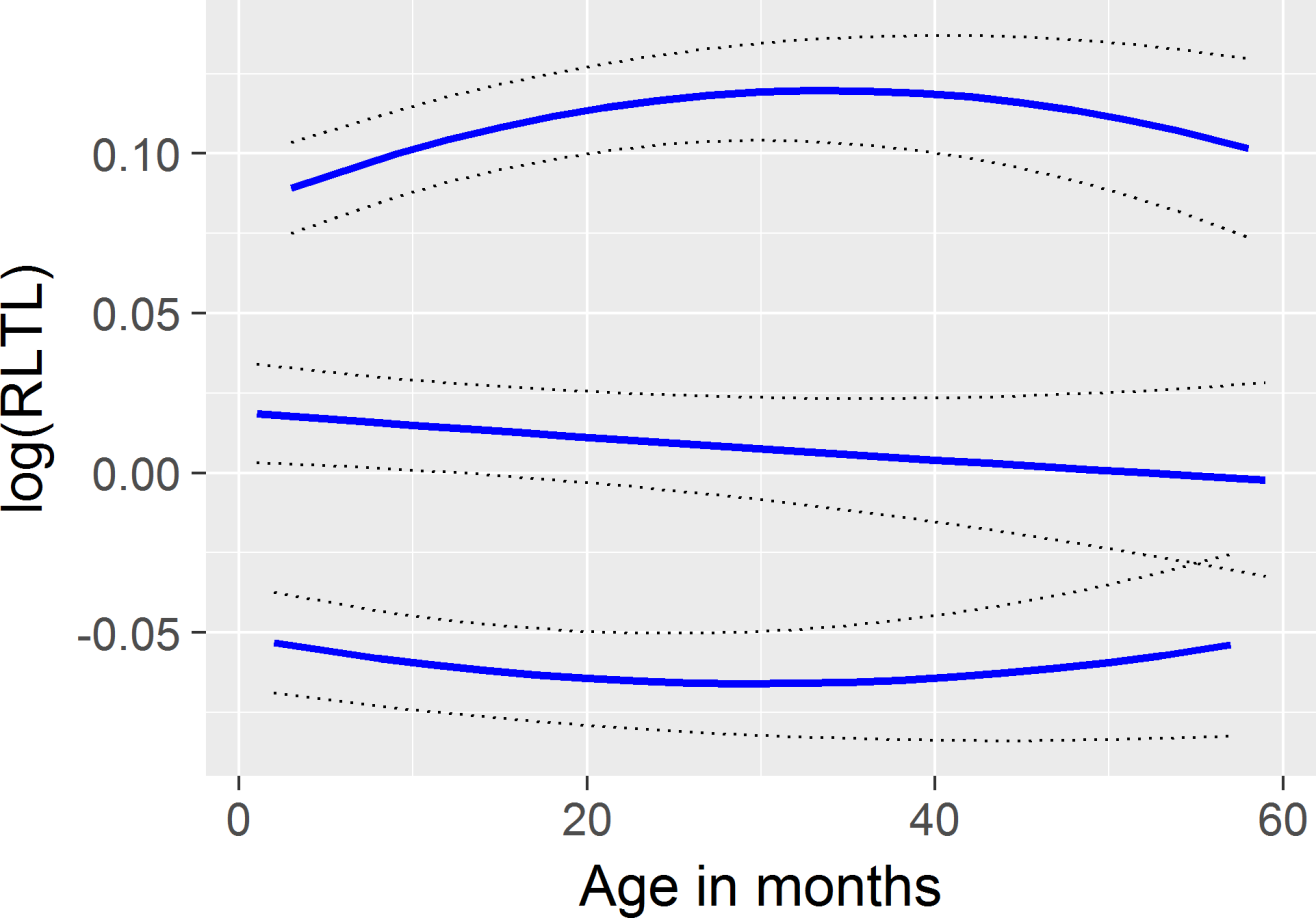
Examples for three individual animal RLTL curves (blue lines) with standard error (black, dotted lines), expressed as deviation from the fixed curve. Animals were chosen randomly to illustrate the variability between individual curves.

Examples of individual animal RLTL curves are shown in Fig 2. These curves illustrate the change in RLTL with age. The intercept, amount and direction of individual RLTL profiles varied considerably and significantly among the animals in the study (Fig 2). The calculation of eigenvalues revealed that the majority of the difference between individual animal profiles is explained by differences in the intercept (94.7%) while 5.3% are due to different shapes of the curves. Eigenfunctions are shown in S1 File.

**Fig 3:**
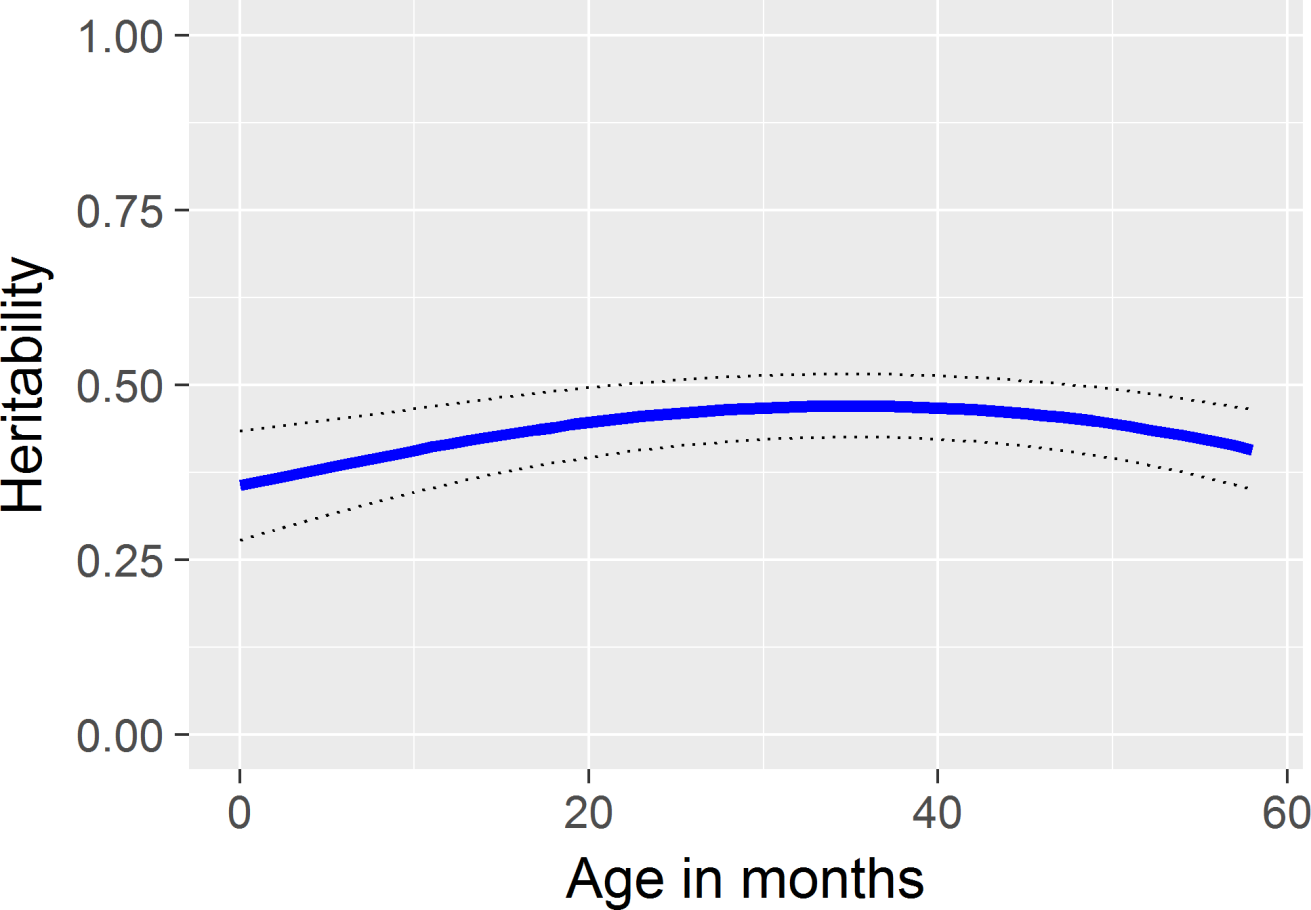
Heritability estimate of RLTL by month of age; standard errors in dotted lines (SE = 0.045–0.078).

Monthly heritability estimates for RLTL ranged from 0.356 to 0.470 (SE = 0.045–0.104) and were slightly higher between 20 and 50 months of age than in the beginning of life or at older ages. Considering the SE, heritability estimates remained relatively stable over life (Fig 3).

**Fig 4:**
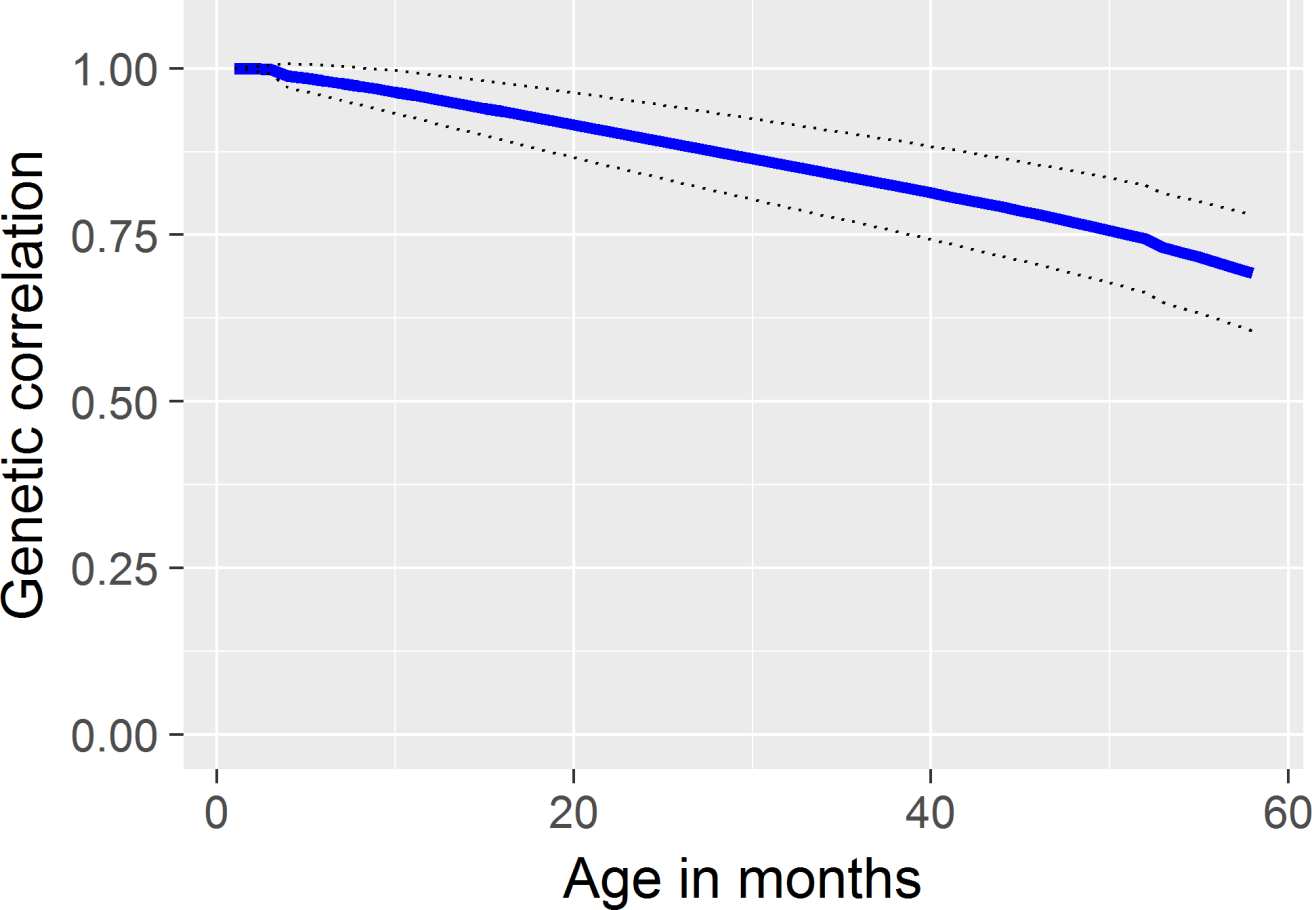
Genetic correlation of RLTL measurements at birth with measurements in later life. standard errors in dotted lines (maximal SE = 0.087).

The genetic correlation between RLTL measurements at birth and at different stages of the animals’ lives are shown in Fig 4. As expected, correlations were very high between RLTL at birth and neighbouring ages but decreased as the interval between the two measurements increased. The minimum correlation was 0.693.

### Analysis of the association of RLTL dynamics with productive lifespan

**Fig 5:**
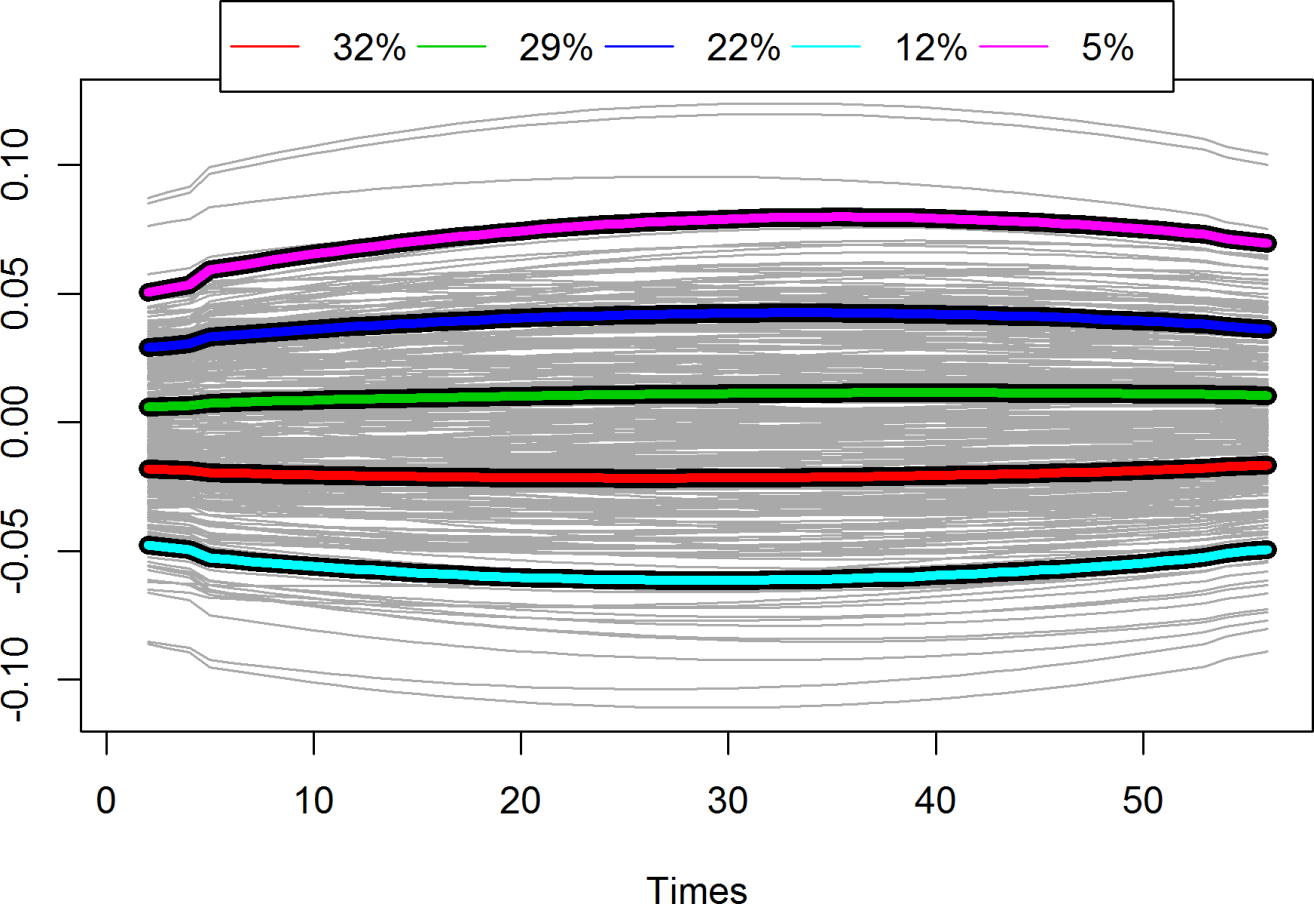
Individual RLTL profiles (grey) and five cluster curves. Of all animals 31% shortened their RLTL slightly in early life (red curve), 30% maintained their RLTL over life (green curve), 22% showed mild elongation in early life (blue curve), 12% more obvious elongation (pink curve) and 4% more obvious telomere attrition (cyan curve).

Productive lifespan ranged from 17 to 2,823 days (mean = 1,477 days, sd = 76.97 days). To test the association between RLTL profiles (intercept and shape) and productive lifespan, RLTL profiles were clustered into groups depending on the similarity of their RLTL change pattern. Five clusters were formed to capture no telomere change and mild and moderate changes in both directions early in life (attrition vs. elongation). Animals differed more in their intercept than in their direction and amount of change. Of all animals 32% shortened their RLTL slightly in early life, while 29% did not show obvious RLTL change at all (red curve and green curves respectively in Fig 5). Mild elongation early in life was observed in 22% (blue curve in Fig 5). More obvious attrition and elongation early in life was observed in 12% and 5% of the animals, respectively (cyan and pink curves in Fig 5).

**Fig 6:**
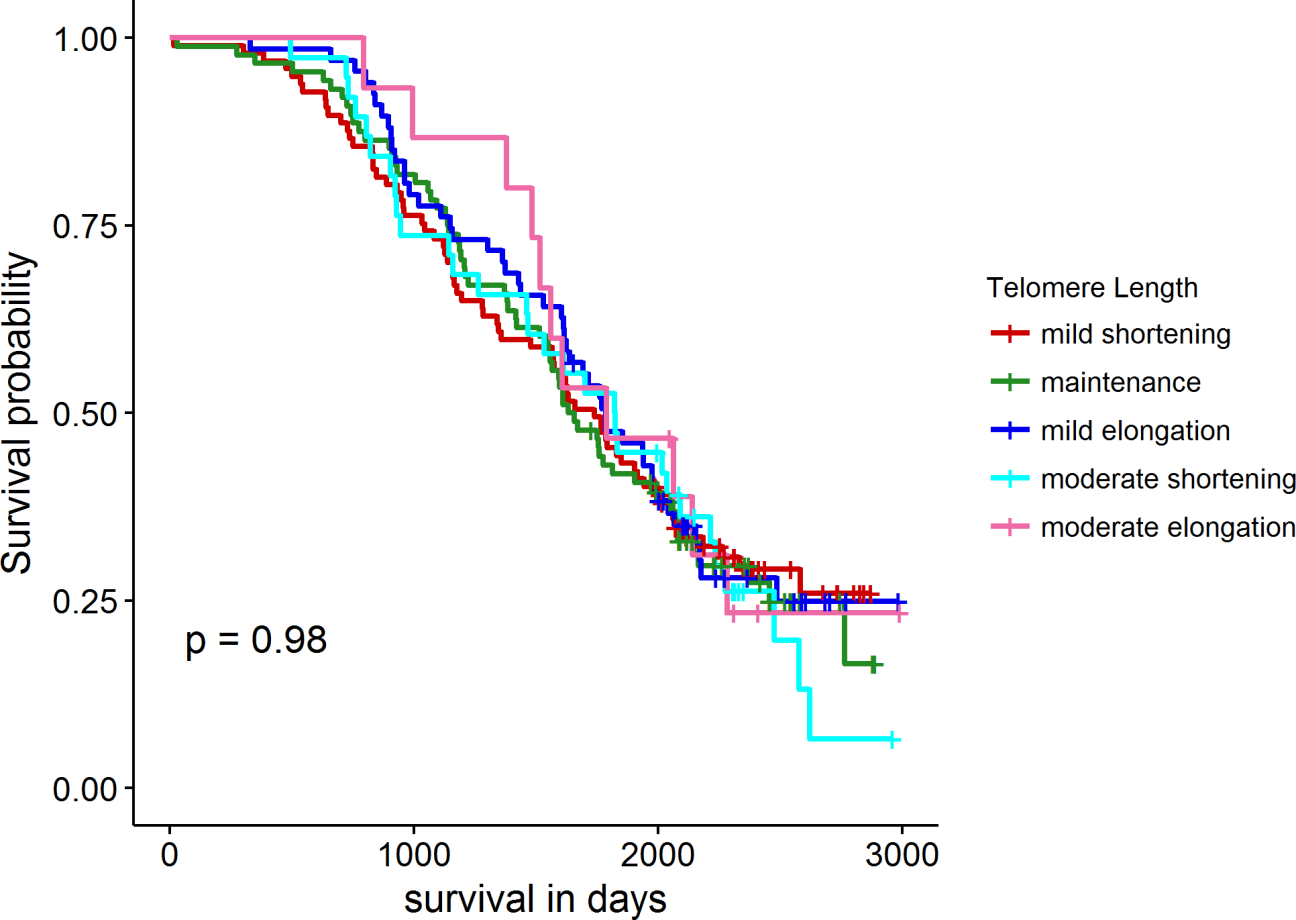
Survival probability of different RLTL profile cluster groups. Colours correspond to colours in Fig 5.

The Cox proportional hazard analysis revealed that there was no significant relationship between RLTL profile cluster and productive lifespan (p = 0.97) which is visualised in Fig 6.

## Discussion

This is the first study exploring individual RLTL profiles of farm animals across time and the largest longitudinal telomere study outside the human literature so far. Our results suggest that individual cattle differ in their RLTL dynamics over life. Although most of the difference between animals is explained by a different average RLTL (intercept) (94.7%), a small proportion is due to different shapes of RLTL profiles (5.3%). This is an important observation that justifies the further investigation of differences in telomere profiles in association with traits of interest such as health, fertility and mortality. The only other study we are aware of that used random regression models for the analysis of longitudinal telomere data did not report a significant difference in telomere dynamics among Seychelles warblers [18], which might have been due to the relatively small sample size of that study.

At a population level RLTL shortened in the beginning of life. The fixed curve calculated in the present study suggests an average RLTL increase later in life. However, this is probably due to the symmetry of a quadratic function and might not reflect biological changes. Therefore, we argue that at a population level telomeres shorten in the beginning of life and remain relatively stable thereafter. Some previous longitudinal studies in baboons and birds support these results, though they did not use random regression models for their analyses [60,61][60,61]. A study in humans found that the early life telomere attrition was followed by a plateau with no telomere change and by a second decline in telomere length as adults grew older [62][62]. It is possible that our study did not include animals that were old enough to show that second decline.

In the present study we report the first heritability estimates for telomere length across all species that were calculated using random regression models. Random regression model estimates do not only inform about the proportion of the variance that is due to additive genetic effects, they also demonstrate how this proportion might change over time. It is known that telomere length is affected by many different genes [22–26]. Epigenetic changes to the genome can alter the translation of genes with ageing [63,64]. If regulatory genes for RLTL were activated or silenced in an unbalanced manner with ageing, heritability estimates for RLTL might change considerably. However, in the present study we show that heritability estimates for bovine RLTL are not only relatively high (0.36 to 0.47; SE = 0.05–0.10) they are also relatively stable (Fig 3). This means that RLTL at all ages could be influenced by breeding programmes. Heritability of telomere length estimated with relatively simpler models has been reported before in humans (0.39–0.82) [27–31], sand lizards (0.52) [32] and kakapos (0.42–0.77)[33].

Within an animal, the genetic correlation between consecutive RLTL measurements decreased as the time interval between measurements increased. This suggests that RLTL might be under different genetic control at different life stages. As mentioned before, epigenetic changes during ageing [63,64] might inhibit or promote genes that play a role in telomere maintenance. Also, telomeres have been reported to have regulatory functions themselves that act on genes in their close proximity and even in further distance [65–67]. For example, long telomeres form bulky structures that can inhibit transcription of genes in their neighbourhood. When telomeres shorten they unfold and enable the expression of those genes. This is known as telomere positioning effect [65]. Also, shelterin can act as transcription factors and thus regulate gene expression [68].

Not much is known about telomere length and its association with productive lifespan in cattle so far. In cross-sectional studies bovine telomere length declines with age and during the lactation period [35,69,70]. A single study found that animals with shorter telomeres were more likely to be culled within the next year [35]. In the present study we did not find a significant relationship between telomere dynamics and productive lifespan in cattle. Dairy cattle rarely live until their physiological end of life but are usually culled for fertility, productivity or health reasons. In the introduction we argued that productive lifespan was still biologically meaningful, because animals are not randomly selected for culling. However, the relationship between productive lifespan and RLTL might be different than these relationships in humans or natural animal populations. Also, a relationship between RLTL and productive lifespan in dairy cattle might be there if RLTL change was examined in a different way. RLTL dynamics might be too pulsatile to be exactly described by random regression models. Future studies are required to investigate the best way to analyse longitudinal datasets that include more than two RLTL measurements per animal. While current results did not show a significant correlation between RLTL and productive life at phenotypic level, a further study examining genetic correlation between the two traits is of high interest as it may provide a different result.

## Supporting information

S1 File Complementary information. (DOCX)
