# Supplementary material for: Longitudinal changes in telomere length and associated genetic parameters in dairy cattle analysed using random regression models
Source: PLoS One. 2018 Feb 13;13(2):e0192864. doi: 10.1371/journal.pone.0192864 (PMC5811042; doi:10.1371/journal.pone.0192864)
Supplement: S1 File — (DOCX) [file pone.0192864.s001.docx]

Supplementary file 1

# Longitudinal changes in telomere length and associated genetic parameters in dairy cattle analysed with random regression models

**Authors & Affiliations:**

Luise A. Seeker^1,2^*, Joanna J. Ilska^1^, Androniki Psifidi^2,3^, Rachael V. Wilbourn^4^, Sarah L. Underwood^4^, Jennifer Fairlie^4^, Rebecca Holland^4^, Hannah Froy^4^, Ainsley Bagnall^5^, Bruce Whitelaw^2^, Mike Coffey^1^, Daniel H. Nussey^4^ & Georgios Banos^1,2^

^1^ Animal & Veterinary Sciences, SRUC, Roslin Institute Building, Easter Bush, Midlothian, UK

^2^ The Roslin Institute and Royal (Dick) School of Veterinary Studies, University of Edinburgh, Easter Bush, Midlothian, UK

^3^ Royal Veterinary College, University of London, Hatfield, UK

^4^ Institute of Evolutionary Biology, School of Biological Sciences, University of Edinburgh, Edinburgh, Midlothian, UK

^5^ SRUC Crichton Royal Farm, Glencaple Road, Dumfries, UK

*corresponding author:

Luise.seeker@sruc.ac.uk
+447591133397

### Model building

We evolved the mixed models used in Seeker et al. (2017, in review) into random regression models. The initial model used in the previous study included the animal identity as a random effect and the following fixed effects: age group of the animal (younger or older than two months) birth year, qPCR plate on which the particular sample was measured, and the qPCR row. For the genetic analysis of RLTL in the present study we also added the genetic group of the animal as an additional fixed effect. For random regression analysis, the time measurements (age) were transformed to a continuous scale (as age in months) to facilitate the interpretation of results.

The significance of the animal identity as random effect was tested by comparing Akaike information criterion (AIC) of a model that only included fixed effects with the model that also included animal identity. The delta AIC was 204.97 indicating that the random effect is statistically highly significant (Table S1). This implies that animals differ in their intercepts and thus in their average RLTL over life. Next, we added pedigree information to the animal identity and compared AIC values with the former model. The pedigree information improved the fit further (delta AIC= 55.46) (Table S1). We fitted the permanent environment as an additional random effect which seemed to have a marginally significant effect on the model fit (delta AIC = 2) (Table S1). However, the effect size of the permanent environment effect was marginal with a relatively large standard error (permanent environment effect variance =0.2 E-09, SE - standard error not estimable, compared to an additive genetic variance= 0.1 E-02, SE =1.9 E -04). We fitted the permanent environment effect to the final random regression model which caused a failure of convergence. Because of the minimal effect size of the permanent environment effect it was judged to be negligible in the present study and therefore removed from further analyses.

Table S1: Comparison of models including different random effects. Fixed effects in the model were: Age in months, genetic group, birth year, qPCR plate and qPCR row. AIC – Akaike information criterion, logL – log-likelihood at convergence, logLratio – twice the difference in logL between the models.

| Model | random effect | AIC | compared with | delta AIC | LogL | logLratio |
| --- | --- | --- | --- | --- | --- | --- |
| Model 0 | / | -5639.91 | model 1 | 204.97 | 2820.96 | 206.96 |
| model 1 | animal identity | -5844.88 | model 2 | 55.46 | 2924.44 | 55.46 |
| model 2 | additive genetic effect | -5900.34 | model 2 | 0 | 2952.17 | 0 |
| model 3 | additive genetic effect + permanent environment | -5898.34 | model 2 | 2 | 2952.17 | 0 |

Next, we added orthogonal Legendre polynomials of increasing order to the fixed effect of age in months and determined the best fitting order by comparing AIC values. A cubic function of age fitted the data best (Table S2). However, when the same order of Legendre polynomial was fitted to the random effect of the animal identity, the model failed to converge. Therefore, the model was simplified by using a quadratic polynomial for both the fixed and the random effect which allowed convergence.

Table S2: Comparison of models including different orders of Legendre polynomials fitted to the fixed effect of age in months. Other fixed effects in the model were: genetic group, birth year, qPCR plate and qPCR row while animal identity with associated pedigree information (additive genetic effect) was fitted as a random effect

| Model | Order of Legendre Polynomial | AIC | delta AIC |
| --- | --- | --- | --- |
| model 1 | NULL | -5900.34 | 74.66 |
| model 2 | linear | -5907.18 | 67.82 |
| model 3 | quadratic | -5963.41 | 11.59 |
| model 4 | cubic | -5975 | 0 |
| model 5 | quartic | -5970.12 | 4.88 |

The inclusion of the quadratic polynomial to the animal identity (random effect) was statistically significant (delta AIC = 3.24). This implies that animals differ in their telomere length profiles and thus in their direction and amount of RLTL change. The quadratic polynomial also described individual deviations from the fixed curve better than a linear function (delta AIC = 2.04) (Table S3).

Table S3: Comparison of different Legendre polynomials fitted to the additive genetic effect of the animal identity. Other fixed effects in the model were age in months (with fitted polynomial), genetic group, birth year, qPCR plate and qPCR row.

| Model | Legendre polynomial (fixed) | Legendre polynomial (random) | AIC | compared to | delta AIC | LogL | logLratio |
| --- | --- | --- | --- | --- | --- | --- | --- |
| model 8 | cubic | cubic | not converged | / | / | / | / |
| model 9 | quadratic | quadratic | -5966.65 | model 9 | 0 | 2990.32 | 0 |
| model 10 | quadratic | linear | -5964.58 | model 9 | 2.07 | 2986.29 | 8.06 |
| model 11 | quadratic | NULL | -5963.41 | model 9 | 3.24 | 2983.7 | 13.24 |

Next, we tested in the random regression model if a heterogeneous variance structure improved the model fit further. First we considered four and then two different variance groups (Table S4).

Table S4: Testing a heterogeneous variance for 1) four different age groups 2) two different age groups; $\boldsymbol{\sigma}^{\mathbf{2}}$ – the residual variance estimate; SE – standard error

|  | age group | $\sigma^{2}$ | $\sigma^{2}$/SE | SE | N |
| --- | --- | --- | --- | --- | --- |
| 1) | 0-12 months | 2.57x10^-3^ | 11.2 | 2.29 x10^-4^ | 465 |
|  | 13-24 months | 1.96 x10^-3^ | 8.55 | 2.30 x10^-4^ | 255 |
|  | 25- 40 months | 2.35 x10^-3^ | 10.6 | 2.22 x10^-4^ | 368 |
|  | > 40 months | 2.05 x10^-3^ | 7.86 | 2.61 x10^-4^ | 240 |
|  |  |  |  |  |  |
| 2) | < 2 months | 2.37 x10^-3^ | 8.17 | 2.90 x10^-4^ | 310 |
|  | > 2 months | 2.27 x10^-3^ | 17.9 | 1.27 x10^-4^ | 1018 |

Differences in the residual variance between considered age groups were not statistically significant (p=0.246 and p= 0.721 for four and two age groups, respectively). Models with homogeneous and heterogeneous variance structures were also compared based on their AIC values. The difference between a homogeneous variance structure and two variance groups was not statistically significant (delta AIC = 1.89), while the homogeneous variance structure was a better fit than four variance groups (delta AIC= 2.09). Therefore, a homogeneous variance structure was assumed in all following models.

### Calculation of genetic parameters

The phenotypic variance (V_P_) for each month equalled V_A_ + V_E_, where V_E_ = environmental variance and V_A_=additive genetic variance. The heritability (h^2^) was calculated for each month as follows (Falconer & Mackay 1996):

$h^{2}=\frac{V_{A}}{V_{P}}$ (S 1)

The standard error (SE) of h^2^ was calculated using following formula (Gilmour et al. 2009):

$SE= \sqrt{(h^{2})^{2}*(\frac{var\left( V_{A} \right)}{V_{A}^{2}}+ \frac{var\left( V_{P} \right)}{V_{P}^{2}}-2* \frac{cov\left( V_{P}, V_{A} \right)}{V_{A}V_{P}})}$ (S 2)

Genetic covariances between different ages in months were calculated using equation (3) in the manuscript except that $\varphi'$ denoted the transpose vector including the residuals for each polynomial order for a different month than $\varphi$. The genetic correlations between RLTL in different months were then calculated using following formula (Falconer & Mackay 1996):

$r_{A}=\frac{{cov}_{xy}}{\sqrt{{var}_{x}{var}_{y}}}$ (S 3)

where cov_xy_ was the genetic covariance between months x and y and var_x_ and var_y_ were the corresponding genetic variance estimates.

Eigenfunctions were calculated by multiplying a matrix containing eigenvectors with a matrix containing Legendre polynomial residuals for each order of the polynomial function and each month. Following formula was used:

$\psi_{i}(x)=\sum_{j=0}^{p-1} \left[ k_{\psi_{i}} \right]_{j}\Phi_{j}(x)$ (S4)

Where $\left[ k_{\psi_{i}} \right]$ is j^th^ element of the i^th^ eigenvector K, $\Phi$ is the jth polynomial of the order p and x is the age in months.


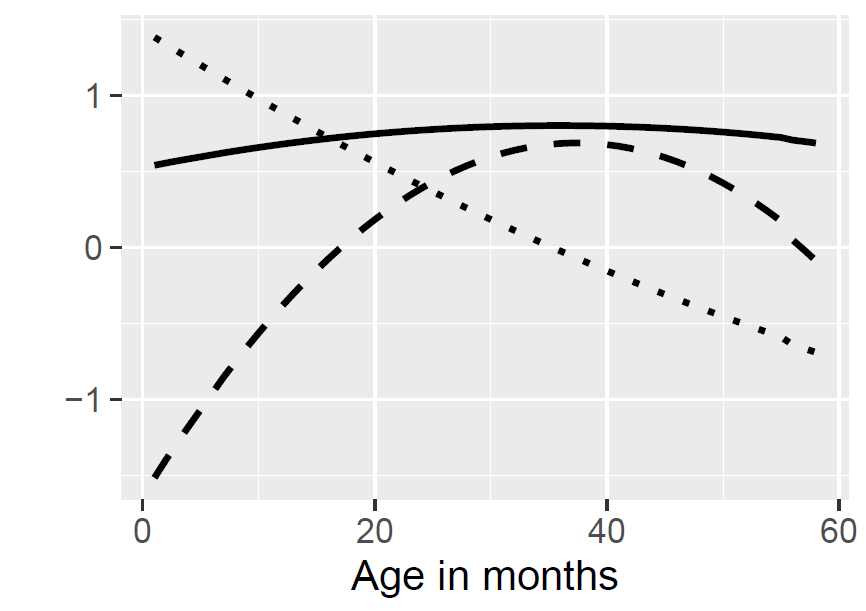


Figure S1: Eigenfunctions (y-axis, unitless) over age in months associated with the largest (intercept; solid line), middle (linear; dotted line) and smallest (quadratic; dashed line) eigenvalue.

### Output of the final random regression model

Table S5: Significance of fixed effects in the final random regression model. A quadratic Legendre polynomial was fitted to age in months. * Significant at p=0.05, ** significant at p = 0.01, *** significant at p= 0.001.

| Source of variation | F statistic | p-value |  |
| --- | --- | --- | --- |
| Intercept | 66.01 | 0.001 | ** |
| Genetic group | 0.02 | 0.871 |  |
| age in months | 16.99 | 0.023 | * |
| Birth year | 1.76 | 0.107 |  |
| qPCR row | 34.53 | <0.001 | *** |
| qPCR plate | 5.98 | <0.001 | *** |
